# MCM4 Is a Novel Biomarker Associated With Genomic Instability, BRCAness Phenotype, and Therapeutic Potentials in Soft-Tissue Sarcoma

**DOI:** 10.3389/fcell.2021.666376

**Published:** 2021-06-10

**Authors:** Qi Liu, Qiyuan Bao, Yiqi Xu, Yucheng Fu, Zhijian Jin, Jun Wang, Weibin Zhang, Yuhui Shen

**Affiliations:** ^1^Department of Orthopedics, Ruijin Hospital, Shanghai Jiao Tong University School of Medicine, Shanghai, China; ^2^Shanghai Institute of Orthopedics and Traumatology, Ruijin Hospital, Shanghai Jiao Tong University School of Medicine, Shanghai, China

**Keywords:** mini-chromosome maintenance protein 4 (MCM4), soft-tissue sarcoma, liposarcoma, genome instability, BRCAness phenotype

## Abstract

Soft-tissue sarcoma (STS) is represented by a heterogeneous group of rare malignancies with various molecular oncogenesis. Therapies targeting DNA repair pathways in STS have achieved minimal progress, potentially due to the lack of molecular biomarker(s) beyond the histology subtype. In this report, we comprehensively analyzed the expression profiles of 100 liposarcomas (LPSs), the most common STS subtype, in comparison with 21 adipose tissues from multiple GEO datasets to identify the potential prognostic and therapeutic biomarker for LPS. Furthermore, we investigated TCGA database, our archived tumor samples, and patient-derived tumor cell cultures (PTCCs) as a validation. We identified a total of 69 common differentially expressed genes (DEGs) among public datasets, with mini-chromosome maintenance protein 4 (MCM4) identified as a novel biomarker correlated with patients’ clinical staging and survival outcome. MCM4-high expression LPS was characterized by MCM4 copy number increase, genomic instability, and BRCAness phenotype compared with the MCM4-low expression counterpart. In contrast, the mutational and the immune landscape were minimally different between the two groups. Interestingly, the association of MCM4-high expression with genomic instability and BRCAness were not only validated in LPS samples from our institution (*n* = 66) but also could be expanded to the pan-sarcoma cohort from TCGA database (*n* = 263). Surprisingly, based on four sarcoma cell lines and eight PTCCs (three LPS and five other sarcoma), we demonstrated that MCM4 overexpression tumors were therapeutically sensitive to PARP inhibitor (PARPi) and platinum chemotherapy, independent of the histology subtypes. Our study, for the first time, suggested that MCM4 might be a novel prognostic biomarker, associated with dysregulated DNA repair pathways and potential therapeutic vulnerability in STS.

## Introduction

Soft-tissue sarcoma (STS) is represented by a heterogeneous group (>70 subtypes) of rare malignancies with a variety of molecular oncogenesis. The metastasis rate of STS in patients with intermediate- or high-grade tumors that are large and deeply seated to the fascia is approximated 50% despite local curative therapy, leading to dismal survival outcome. Currently, the prognostic and predictive biomarker(s) beyond histology-based classification is still lacking.

For example, liposarcoma (LPS) is one of the most common types of STS in the extremities and retroperitoneum with a variety of molecular pathogenesis (Crago and Brennan, 2015). Studies have shown that the primary pathological assessment of LPS results in a 25% misclassification of the histologic subtypes, indicating a pathological and morphological continuum of LPS tumor cells (de Vreeze et al., 2010). Furthermore, due to the inter-tumor heterogeneity, the biological behavior of the same LPS tumor could be drastically varied from proportion to another (Swanton, 2012; Bill et al., 2016). It was estimated that 20–40% of relapsed well-differentiated LPS (WDLS) could be identified with a dedifferentiated LPS (DDLS) component (Singer et al., 2003; Fabre-Guillevin et al., 2006). In contrast, tumor cells of different subtypes of LPS could also share common signaling pathways (Bill et al., 2016), epigenetic aberration (Chen et al., 2019), and intra-tumoral immune microenvironment (Tseng et al., 2015). Therefore, patient stratification based on histology alone is insufficient for the prognostication and management of sarcoma. Unfortunately, the molecular biomarkers for most of the STS have been minimally improved over the past decades (Patel et al., 2017). Therefore, novel histology-independent biomarker(s) for tailored prognosis and therapeutic regimen is as-yet to be exploited in LPS as well as in other histology subtypes.

**FIGURE 1:**
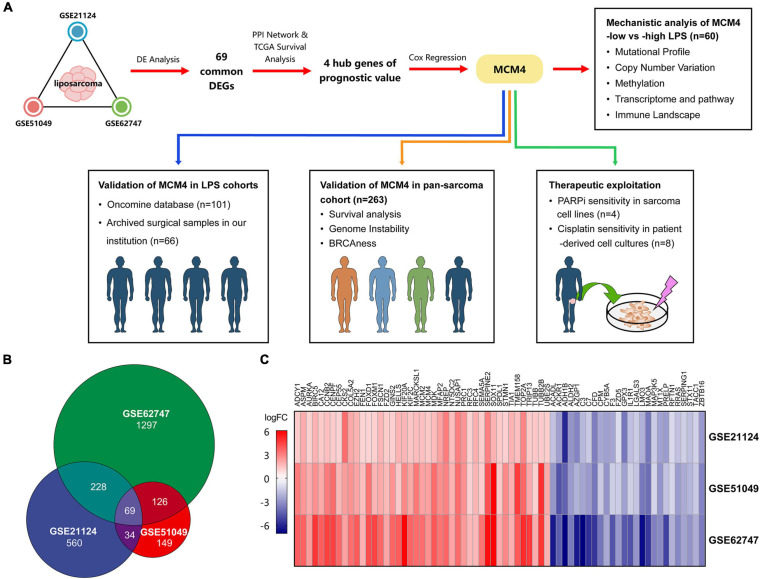
The overall study design and the identification of the potential biomarkers. The schematic graph represents the overall design of the study. Mini-chromosome maintenance protein 4 (MCM4) was prioritized from multiple Gene Expression Omibus (GEO) datasets, and validated in liposarcoma (LPS) and pan-sarcoma cohorts. The therapeutic potential of the MCM4-high expression subgroup was explored in cell lines and patient-derived tumor cell cultures **(A)**. A total of 69 common differentially expressed genes (DEGs) among three datasets of LPS **(B)**, with the corresponding gene expression level shown in the heatmap **(C)**.

In this report, we comprehensively analyzed the transcriptome of 100 LPS and 21 adipose tissue samples from multiple Gene Expression Omnibus (GEO) datasets, and identified mini-chromosome maintenance protein 4 (MCM4) as a novel biomarker associated with patient prognosis, as well as the genomic instability and BRCAness phenotype. We then investigated the MCM4 expression profiles from our archived tumor samples, nine histology subtypes of STS in TCGA, and patient-derived tumor cell cultures (PTCCs) as a validation (Figure 1A).

## Materials and Methods

### Gene Expression Omnibus Datasets and Microarray Data Analysis

To study the gene expression profiles, we obtained three LPS cohorts from the GEO database (Supplementary Table 1): (1) GSE21124 (89 LPS and 9 adipose tissues), (2) GSE51049 (4 LPS and 4 adipose tissues), and (3) GSE62747 (7 LPS and 8 adipose tissues). The online tool GEO2R (Davis and Meltzer, 2007) was used to screen for the differentially expressed genes (DEGs) between cancer and normal samples, according to the criteria of false discovery rate (FDR) < 0.05 and | fold-change (FC)| ≥ 2. The results were then overlapped to identify the common DEGs among three cohorts (Figures 1A,B). For multiple probes mapping to the same gene, we exhibited those with the max | log2FC| in the heatmap. Besides, to explore the gene expression between cell lines with different therapeutic sensitivity, we downloaded the raw RNA-seq data of sarcoma cell lines from GSE76981, comprising four STS cell lines (HT1080, SW684, DMR, and 402.91) and two bone sarcoma cell lines (TC106 and SJSA1).

### Prioritization of the Prognostic Biomarker of Liposarcoma

To prioritize the gene of interest from common DEGs, a protein–protein interaction network was constructed using STRING database (Supplementary Table 1). The Molecular Complex Detection (MCODE) app in Cytoscape software v3.7.1 (confidence score ≥ 0.4) (Shannon et al., 2003) was used to remove the separated nodes in network, thereby leaving the key hub genes.

Using the clinical data of 60 patients with LPS in GDC TCGA-SARC cohort (Supplementary Table 1), the hub genes were then assessed by univariate and multivariate Cox regression analysis via the “survival” R package to prioritize the gene with the greatest prognostic significance. In univariate Cox regression analysis, we computed the hazard ratio of the hub genes contributing to the worse survival outcome (death). The hazard ratio is defined as the ratio of (hazard rate in study group)/(hazard rate in control group), while the hazard rate is the chance of a hazardous event occurring at a given time (Blagoev et al., 2012). The gene expression values were dichotomized according to the median expression into high-expression subset and low-expression subset. Furthermore, to identify the independent prognostic biomarker in LPS, multivariate analysis was performed among genes with hazard ratio > 1 and *p* < 0.05 in the univariate analysis. Results were demonstrated using “forestplot” and “survival” package in R. The receiver operating characteristic (ROC) curves were plotted to predict the 1-, 3-, and 5-year survival of patients based on MCM4 expression, via “survivalROC” package in R.

### Multi-Omics Analysis of MCM4-High Liposarcoma

Multi-Omics data of the aforementioned 60 LPS specimens were obtained from the GDC data portal as well (Supplementary Table 1). LPS were classified into MCM4-high (*n* = 30) vs. MCM4-low (*n* = 30) subset using the median MCM4 expression level as the cutoff. For somatic mutations, we compared the difference of mutation frequencies and tumor mutation burden (TMB) between these two groups, and visualized the results by “maftools” package in R. The TMB was calculated as the total mutation frequency/megabase (Mb) for each sample. Then, we analyzed the association of MCM4 copy number and MCM4 methylation with MCM4 expression. The test results were visualized by “ggplot2” and “ggpubr” package in R. Finally, the transcriptome and immune landscape between the subgroups were compared using Gene Set Enrichment Analysis (GSEA) (Subramanian et al., 2005) (gene set permutations of 1,000 times, *P* < 0.05 and FDR < 0.05) and EPIC software (Racle et al., 2017), respectively. Wilcoxon test was used to compare the difference in immune cell infiltration between two groups.

### Calculation of Genomic Instability and BRCAness

The proportion of the copy number variations (CNVs) across the whole genome (genome-wide CNVs) and weighted genome instability index (wGII) across 22 autosomal chromosomes were measured to estimate the genomic instability of sarcoma (Dewhurst et al., 2014). Moreover, to assess the function loss of homologous recombination (HR) pathway, we calculated the BRCAness (BRCA-like phenotypes shared by non-BRCA-mutated tumors) score on the transcriptome level based on the 60 gene signature (Konstantinopoulos et al., 2010), in addition to the PARP1 expression, which was reportedly associated with HR deficiency and therapeutic efficiency in sarcoma (Pignochino et al., 2017).

### Validation of MCM4 Expression of Liposarcoma and Other Soft-Tissue Sarcoma in Oncomine Database

Oncomine database (Supplementary Table 1) was used to assess the gene mRNA expression for common types of sarcoma and the corresponding normal tissues. In this study, “MCM4” was searched with the following filter criteria: (1) threshold (*P* < 1E–4, FC > 2, gene rank: top 10%), (2) data type: mRNA, (3) analysis type: cancer vs. normal analysis, and (4) cancer type: sarcoma.

### Validation of the MCM4 as a Biomarker in Pan-Sarcoma Cohort

The UCSC Xena database (Supplementary Table 1) was utilized to acquire the Genotype-Tissue Expression (GTEx; Supplementary Table 1) and TCGA gene expression data, so as to explore whether MCM4 transcripts were distinguishable between STS and 36 types of normal tissues (*n* = 8,425). Meanwhile, we used cBioPortal database (Supplementary Table 1) to retrieve the additional clinical information of 263 STS specimens, including cancer subtype classification, MCM4 copy number, metastasis, mitotic count, tumor necrosis rate, and survival outcome, thus, broadening our findings derived from LPS to a wider pan-sarcoma population.

### Immunohistochemistry Validation of the Archived Sarcoma Specimens

As a validation, 66 MCM4 protein expressions from the surgical specimens of lipomatous neoplasms and normal adipose tissues were collected from patients diagnosed at Ruijin Hospital, affiliated to Shanghai Jiao Tong University School of Medicine. Among them were 39 LPS samples (malignant), 22 lipoma samples (benign), and five adipose tissue samples (normal). The pathological analysis was independently done by two expert pathologists, who identified tumor stages and grades according to the AJCC STS s staging system (8th) (Tanaka and Ozaki, 2019). The malignant group comprised 20 cases of WDLS, 9 cases of DDLS, 9 cases of myxoid LPS (MLS), and 1 case of pleomorphic LPS (PLS). All malignant samples were equipped with the information of Ki67 labeling index and S-100.

Paraffin-embedded tissues were cut into slices of 4 μm thickness. After heat-induced antigen retrieval, we incubated sections in a rabbit anti-MCM4 antibody (monoclonal; D3H6N, 1:200; CST) at 4°C overnight. Breast cancer sections with MCM4 expression were used as the positive control, while samples without primary antibody incubation were selected as the negative control. We graded the intensity of nuclear staining for MCM4 (0, no staining; 1, yellow; 2, pale brown; 3, dark brown), and scored the extent of staining based on the rate of the positive cell (0, < 5%; 1, 5–25%; 2, 26–50%; 3, 51–75%; 4, 76–100%). By multiplying the color value with positive cell rate, we got the final IHC score: 0–2 (–), 3–4 (+), 5–8 (++), and 9–12 (+++).

### Establishing Patient-Derived Tumor Cell Cultures From Sarcoma Specimens

Eight STS specimens were collected from the tumor biopsy (the corresponding clinical data was shown in Supplementary Table 2), which were cut into 1–3 mm^3^ pieces after PBS washing. The tissue pieces were digested in 10 mL of Dulbecco’s modified Eagle’s medium (DMEM) supplemented with 10% fetal bovine serum (FBS), 1% penicillin–streptomycin, 5 μg/mL Amphotericin B (V900919, Sigma), and 1 mg/mL collagenase I (17100017, Thermo Fisher Scientific) on a constant temperature (37°C) water bath shaker for 1 h. We collected the digested cells by centrifugation at 1,500 rpm for 5 min. The pellet was then resuspended in 4 mL of fresh cell culture medium and filtered through a 70 μm filter. Afterward, dead or non-adherent cells were removed by medium change after 2 days, and adherent live cells were kept in culture medium. To explore the corresponding tumoral MCM4 expression, adherent cell cultures were digested and centrifugated into pellets, followed by 4% paraformaldehyde fixation and histological study, including HE staining and IHC labeling for MCM4.

### Therapeutic Investigation of MCM4-High Soft-Tissue Sarcoma

PTCCs were treated with DMEM supplemented with 10% FBS and cisplatin (P4394, sigma) at 0.1, 0.5, 1.0, 5.0, and 10.0 μM for 24 h. To confirm the cytotoxicity of cisplatin for sarcoma cells, cell viability was measured by CCK-8 assay (CK04, Dojindo). Then we used built-in equations from Graphpad prism 8.0 (inhibitor vs. normalized response with Variable slope) to assess IC50, and compared the difference of IC50 between two groups via unpaired *t*-test. Also, Western blotting (WB) was performed as previously described (Peng et al., 2020). Briefly, we separated the proteins by 10% SDS-PAGE gel, and transferred them onto the polyvinyl difluoride (PVDF) membranes. The membranes were blocked by 5% bovine serum albumin (BSA) for 2 h at room temperature and incubated with primary antibodies overnight at 4°C. All primary and secondary antibodies can be found in Supplementary Table 3. Additionally, the gene expression profiles of four STS cell lines (Pignochino et al., 2017) were retrieved from GEO database to validate the association of MCM4 expression with therapeutic vulnerabilities.

## Results

### Identifying Common Differentially Expressed Genes in Liposarcoma

LPS is one of the most common types of STS with a rich source of public data. We therefore started by analyzing a total of 121 samples, including 100 LPS and 21 adipose tissues in our study (Figures 1A,B). Based on the criteria of FDR < 0.05 and | FC| ≥ 2, we totally screened 339, 221, and 760 DEGs from GSE21124, GSE51049, and GSE62747 datasets, respectively. Sixty-nine DEGs were commonly found among three datasets, including 43 upregulated and 26 downregulated genes (Figure 1C).

### MCM4-High Expression as a Robust Prognosticator in Liposarcoma Patients

**FIGURE 2:**
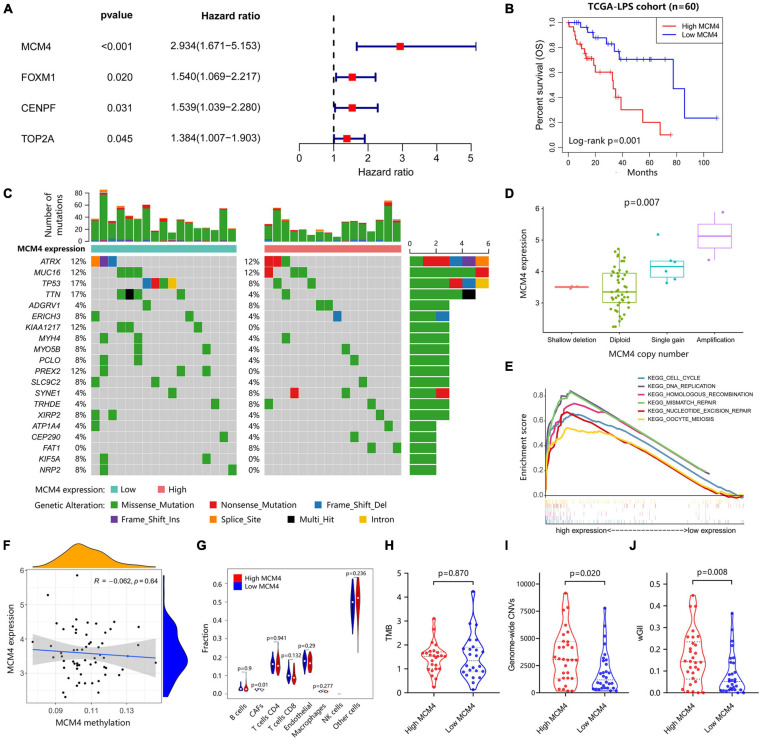
Integrated genomic characterization of the MCM4-high and MCM4-low expression subgroup of LPS in TCGA (*n* = 60). **(A)** The four hub genes were investigated using the univariate Cox-regression analysis. The hazard ratio and the 95% confidence interval of each gene were shown in forest map. **(B)** The overall survival of the MCM4-high subgroup is significantly worse than that of the MCM4-low subgroup in LPS with Log-rank test *p* < 0.001. **(C)** The landscape of somatic mutations between MCM4 high- and low-expression LPS demonstrated recurrent mutations in ATRX, MUC16, TP53, etc. There was no significant difference in the frequency of the somatic mutations between the two groups (*t*-test, *p* > 0.05). **(D)** The MCM4 expression of LPS tumor samples was significantly affected by the copy-number variations of MCM4 (Kruskal–Wallis, *p* = 0.007). **(E)** The KEGG pathways enrichment analysis indicated that the transcriptome of the MCM4-high vs. the MCM4-low subset was different in several pathways, including the cell cycle, DNA replication and multiple DNA damage repair gene sets. **(F–H)** The tumor mutation burden (TMB) (*t*-test, *p* = 0.870), MCM4 methylation (Spearman’s correlation, *p* = 0.640), and immune cell infiltration (Wilcoxon test, *p* > 0.05; except for CAFs) were minimally different between the MCM4-high and MCM4-low LPS. In contrast, the MCM4-high LPS exhibited a higher level of genomic instability than the MCM4-low counterpart, as indicated by genome-wide copy number variations (CNV) burden [**(I)**; Wilcoxon test, *p* = 0.020] as well as the weighted Genome Instability Index score [**(J)**; Wilcoxon test, *p* = 0.008].

Based on the STRING database, we constructed a protein–protein interaction network complex of 48 genes and 285 edges (average local clustering coefficient: 0.579; the enrichment *p* < 1.0e–16) (Supplementary Figure 1A) from the 69 DEGs, resulting in 22 “hub” genes hypothetically of great importance in LPS (Supplementary Figure 1B). We then asked whether these hub genes correlated with the patients’ survival outcome in LPS. Interestingly, in univariate Cox regression analysis, a total of four hub genes (CENPF, FOXM1, MCM4, and TOP2A) were found to have prognostic significance in terms of the overall survival, with MCM4 associated with the greatest hazard ratio of 2.934 (95% CI, 1.671–5.153, *p* < 0.001) (Figure 2A). Multivariate analysis further resulted in MCM4 as the only independent risk factor in LPS. After dichotomizing 60 LPS cases into MCM4-high (*n* = 30) and low (*n* = 30) expression subsets, we found that the overall survival was drastically worse in MCM4-high patients than the MCM4-low counterpart (Figure 2B). The ROC curve suggested a high predictive value of MCM4 for the 1–, 3–, and 5-year survival, respectively (Supplementary Figure 1C).

### Integrated Multi-Omic Comparison of MCM4-High vs. MCM4-Low Liposarcoma

Whether MCM4-high LPS represents a mechanistically distinct entity with its own therapeutic potential remains an open question. We, therefore, investigated the genomic, epigenomic, transcriptomic, and immunological profiles between MCM4-high and MCM4-low LPS from TCGA cohort. As previously reported (Cancer Genome Atlas Research Network, 2017), recurrent mutations were found in TP53, ATRX, MUC16, etc., in both MCM4-high and -low subsets. However, we did not notice any statistical significance in any mutated genes (Figure 2C), total mutational burden (Figure 2H) or MCM4 gene methylation level (Figure 2F) between the two subsets (*p* > 0.05). The immune cell infiltration (Figure 2G and Supplementary Figures 2A,B) and immune checkpoints molecules expression such as PD-1, LAG3, CTLA4, etc. (Supplementary Figure 2C) were also minimally different between the MCM4-high and MCM4-low LPS. In contrast, copy number alteration analysis demonstrated that MCM4 expression was significantly affected by gene copy number (*p* = 0.007; Figure 2D).

The dysfunction of MCMs has been associated with double-strand DNA unwinding, DNA replication control, and DNA damage repair in several epithelial cancers (Yu et al., 2020). Consistently, GSEA demonstrated that the MCM4-high subgroup demonstrated an overexpression of cell cycle, DNA replication, as well as the HR pathways (Figure 2E and Supplementary Figure 3). In parallel, we observed that MCM4-high LPS more frequently harbored copy number loss in genes of HR pathway (Supplementary Figure 4). Furthermore, the MCM4-high LPS exhibited a higher level of genomic instability than the MCM4-low counterpart, as indicated by genome-wide CNV burden (*p* = = 0.019; Figure 2I) as well as the wGII score (*P* = 0.008; Figure 2J). In contrast, neither MCM4 copy number (*P* = 0.268) nor CNV burden (*P* = 0.636) was predictive of the patients’ overall survival (Supplementary Figures 1D,E).

### Validation of MCM4 Expression With Oncomine Database

**FIGURE 3:**
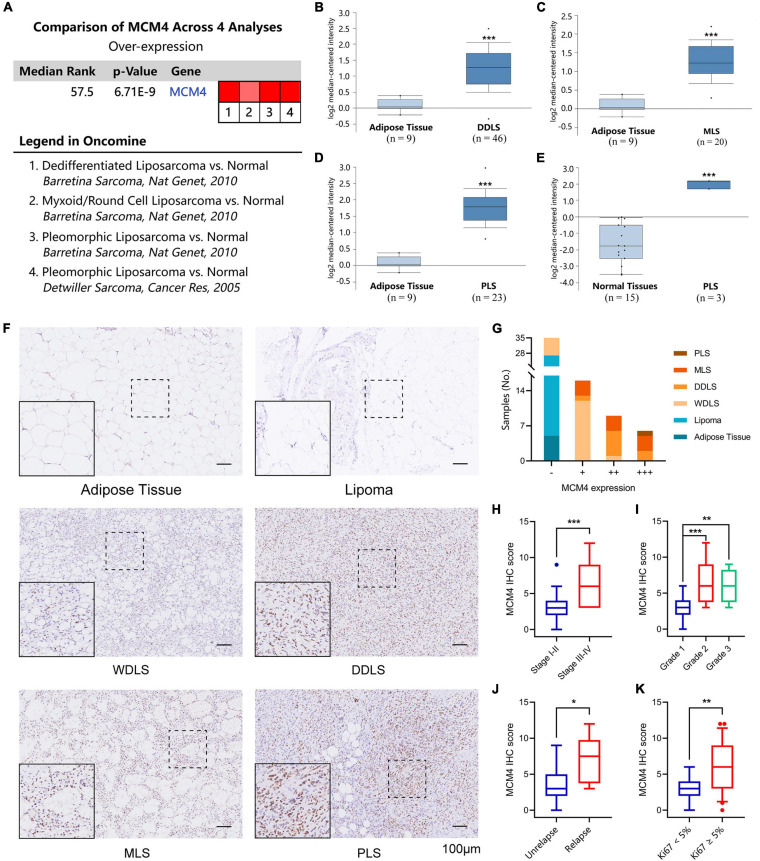
Validation of MCM4 as a biomarker of LPS by Oncomine database and archived surgical specimens. A total of four registries supporting the high tumoral expression of MCM4 vs. normal, while no studies supported the MCM4-high expression in normal tissues **(A)**. MCM4 was found to be consistently overexpressed in dedifferentiated LPS (DDLS) **(B)**, Myoxoid LPS (MLS) **(C)**, and Pleomorphic LPS (PLS) **(D,E)** compared to the adipose control. Using 66 archived surgical specimens, we confirmed the overexpression of MCM4 in LPS, but not the adipose tissue or benign lipoma **(F,G)**. MCM4 Immunohistochemistry (IHC) scores significantly correlated with AJCC stage, histological grade, tumor relapse-free survival, and Ki67 index (*t*-test, *p* < 0.05) **(H–K)**. *p < 0.05, **p < 0.01, **p < 0.001,scale bar = 100 um.

By searching a total of 75 significant unique analysis records from the Oncomine database, we noticed four LPS studies supporting the high tumoral expression of MCM4 compared with the normal, while no studies supported the MCM4-high expression in normal tissues (Figure 3A). Specifically, MCM4 was found to be consistently overexpressed in DDLS (222036_s_at), MLS (222036_s_at), and PLS (222036_s_at and 212141_at) compared with the adipose control (Figures 3B–E).

### Validation of MCM4 Signature With Archived Liposarcoma Samples From Our Institution

Next, we performed IHC staining of MCM4 for 66 archived surgical specimens in our institution, including 39 LPS, 22 lipomas, and 5 normal adipose tissues (Figure 3F). We found that MCM4 expression was positive in 79.5% of the LPS specimens across multiple histology subtypes, but not in the benign or normal tissues (Figure 3G and Table 1). For LPS, MCM4 was significantly correlated with AJCC stage, histological grade, tumor relapse-free survival, and Ki67 index (*p* < 0.05), but not gender, age, tumor location, etc. (Figures 3H–K and Table 2). These results confirmed that MCM4-high LPS as a potentially aggressive subset with poor clinical prognosis across the histology subtypes.

**TABLE 1:** Mini-chromosome maintenance protein 4 (MCM4) expression in adipose tissues and lipomatous tumors.

Groups	Cases	Low		High		Positive rate (%)	*P*-value
		(–)	(+)	(++)	(+++)		
Malignant	39	8	16	9	6	79.5	<0.001*
Benign	22	22	0	0	0	0	
Normal	5	5	0	0	0	0	

*P < *0.05 was regarded as statistically significant.*

**TABLE 2:** Correlation between MCM4 expression and pathological parameters in liposarcoma (LPS).

Characteristics	Cases	Low		High		High positive rate (%)	*P*-value
		(–)	(+)	(++)	(+++)		
Gender							
Male	24 (61.5%)	3	13	5	3	33.33	0.405
Female	15 (38.5%)	5	3	4	3	46.67	
**Age (years)**							
<60	22 (56.4%)	3	10	6	3	40.91	0.721
≥60	17 (43.6%)	5	6	3	3	35.29	
**Tumor size (cm)**							
≤ 10	7 (17.9%)	2	2	2	1	42.86	0.792
> 10	32 (82.1%)	6	14	7	5	37.50	
**Location**							
Trunk and extremity	30 (76.9%)	5	13	6	6	40.00	0.718
Retroperitoneum	9 (23.1%)	3	3	3	0	33.33	
**Stage**							
I + II	24 (61.5%)	8	12	3	1	16.67	<0.001*
III + IV	15 (38.5%)	0	4	6	5	73.33	
**Grade**							
G1	23 (58.9%)	8	12	3	0	13.64	<0.001*
G2	12 (30.8%)	0	3	4	5	75.00	
G3	4 (10.3%)	0	1	2	1	75.00	
**Relapse^#^**							
No	9 (47.4%)	3	4	1	1	22.22	0.037*
Yes	10 (52.6%)	0	3	2	5	70.00	
**Ki67 labeling index**							
<5%	18 (46.2%)	5	11	2	0	11.11	0.001*
≥5%	21 (53.8%)	3	5	7	6	61.90	
**S-100 expression**							
Negative	9 (23.1%)	2	4	2	1	33.33	0.718
Positive	30 (76.9%)	6	12	7	5	40.00	

*P < *0.05 was regarded as statistically significant. ^#^Due to incomplete follow-up data, 19 samples were included in the tumor relapse comparison.*

### Validation of MCM4 Signature in Pan-Sarcoma Cohort

**FIGURE 4:**
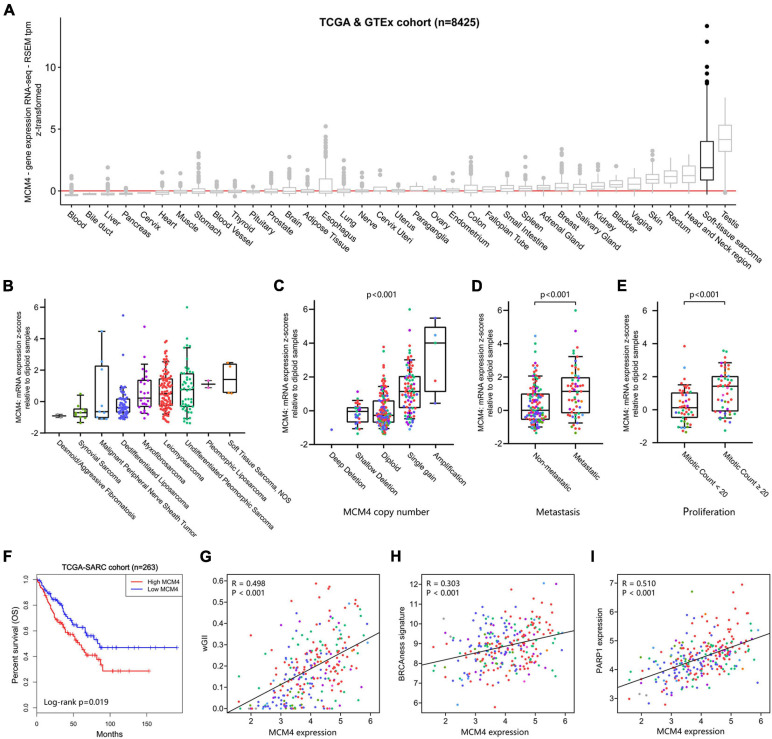
Validation of MCM4 signature in pan-sarcoma cohorts. **(A)** MCM4 is highly expressed in soft-tissue sarcoma and testis, compared with the normal tissues. **(B)** The landscape of MCM4 expression in various soft-tissue sarcoma (STS) subtypes from TCGA database. **(C–F)** MCM4 expression was correlated with MCM4 copy-number (Kruskal-Wallis, *p* < < 0.001, *n* = 255), metastatic state (Wilcoxon test, *p* < 0.001, *n* = 179), proliferation index (Wilcoxon test, *p* < 0.001, *n* = 93), and patient overall survival (Log-rank test, *p* < 0.019, *n* = 263) across multiple histology subtypes in STS. **(G–I)** The tumoral expression of MCM4 was observed to be positively correlated with genome instability (weighted Genome Instability Index (wGII) score; *p* < 0.001, *R* = 0.498, *n* = 263), BRCAness signature (*p* < 0.001, *R* = 0.303, *n* = 263), and PARP1 expression (*p* < 0.001, *R* = 0.510, *n* = 263) in STS, via Spearman’s correlation analysis.

Using the UCSC Xena database (Goldman et al., 2020), we found that MCM4 overexpression was found almost exclusively in STS and testis, but not in other types of normal tissue (Figure 4A). Interestingly, the abundance of different MCM4 transcripts was distinguishable between STS and testis (Supplementary Figure 6C). A survey of all STS registries in Oncomine database confirmed a consistent overexpression of MCM4 not only in LPS but also in leiomyosarcoma, fibrosarcoma, synovial sarcoma, and undifferentiated pleomorphic sarcoma, compared with the normal (Supplementary Figure 5). To explore whether our findings derived from LPS could be broadened to a wider pan-sarcoma population, 263 STS specimens from TCGA-SARC (Cerami et al., 2012), including 104 leiomyosarcoma, 58 DDLS, 49 undifferentiated pleomorphic sarcoma, 25 myxofibrosarcoma, 10 synovial sarcoma, 9 malignant peripheral nerve sheath tumor, 4 sarcoma NOS (not otherwise specified), 2 PLS, and 2 desmoid fibromatosis, were assessed for MCM4 expression (Figure 4B). Surprisingly, the MCM4 expression was also found to be associated with MCM4 copy number (*p* < 0.001; Figure 4C), higher metastasis potential (*p* < 0.001; Figure 4D), tumor mitotic count (*p* < 0.001; Figure 4E), and worse survival outcome (*p* = 0.019; Figure 4F), but not tumor necrosis rate (Supplementary Figure 6A). In parallel with what we found in LPS, the MCM4 expression was positively correlated with genomic instability (wGII score) in STS (*R* = 0.498, *p* < 0.001; Figure 4G). Despite the lack of deleterious mutation in HR pathway (Supplementary Figure 6B), MCM4 overexpression STS also harbored an HR-deficiency (BRCAness) phenotype (*R* = 0.303, *p* < 0.001) as well as PARP1 overexpression (*R* = 0.510, *p* < 0.001) in a histology-agnostic fashion (Figures 4H,I).

### Therapeutic Exploitation of MCM4 as a Predictive Biomarker of Sarcoma

**FIGURE 5:**
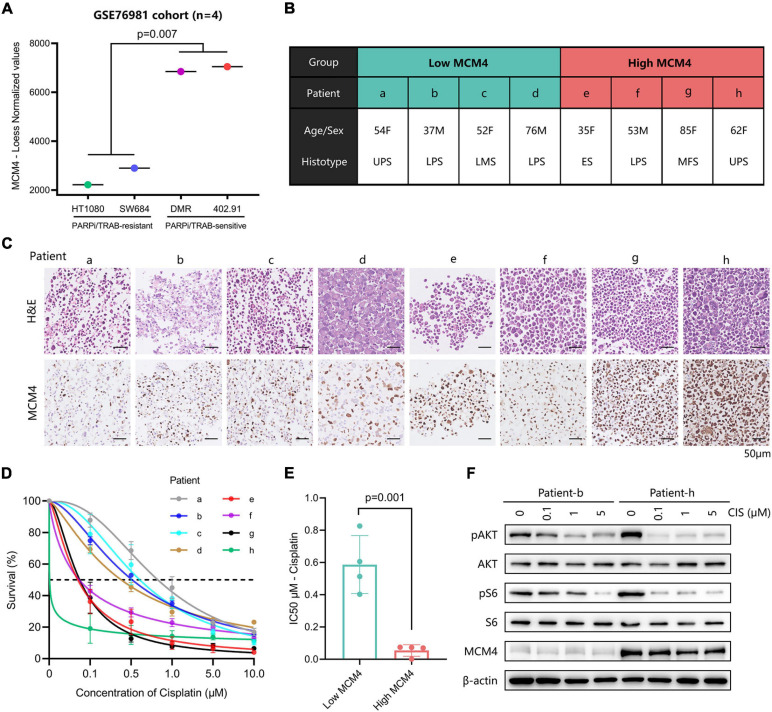
Evaluation of the therapeutic potential in MCM4-high STS cell lines and patient-derived tumor cell cultures (PTCCs). **(A)** By comparing the gene expression profiles of four STS cell-lines, we found that MCM4 was drastically overexpressed in the PARPi/Trabectedin-sensitive cell lines compared with the PARPi/Trabectedin-resistant cell lines (*t*-test, *p* = 0.007, *n* = 4). **(B,C)** Patient-derived tumor cell cultures (PTCCs) were established from the biopsy of eight STS patients, ranked by the corresponding tumoral MCM4 expression. After treated with cisplatin, the MCM4-high PTCCs (patient e–h) demonstrated less cell proliferation **(D)**, with a lower half inhibitory concentration (IC50) than the MCM4-low subset (a–d) (*t*-test, *p* = 0.001, *n* = 8) **(E)**. Additionally, the levels of p-AKT and p-S6 were significantly reduced when cell growth was inhibited **(F)**.

Both of the genomic instability (Andor et al., 2017) and BRCAness (Konstantinopoulos et al., 2010) have been associated with increased sensitivity of tumor to DNA-damaging agents (such as cisplatin) and PARPi. To test this hypothesis in MCM-high STS, we first retrieved the expression profiles of four STS cell lines known to have a distinct vulnerability to PARPi or trabectedin, according to Pignochino et al. (2017). Interestingly, MCM4 was drastically overexpressed in the PARPi/trabectedin-sensitive cell lines compared with the PARPi/trabectedin-resistant cell lines (*p* = 0.007; Figure 5A). Next, PTCCs were established from the biopsy of eight STS patients (Figure 5B) and ranked from a to h according to the MCM4 IHC expression (Figure 5C). After treatment with cisplatin, the MCM4-high PTCCs (e–h) demonstrated inhibited cell proliferation (Figure 5D), with a lower half inhibitory concentration (IC50, 0.001–0.075 μM) than the MCM4-low subset (a–d) (0.403–0.827 μM, *p* = 0.001; Figure 5E). Additionally, the levels of p-AKT and p-S6 were also reduced when cell growth was inhibited (Figure 5F).

## Discussion

DDLS, high-grade MLS, and PLS are high-grade adipose sarcoma with disease-specific survival (DSS) of 44, 74, and 59%, respectively (Dalal et al., 2006). Although rarely metastasizing, WDLS, and low-grade MLS are at high risk of local failure, leading to poor general performance, and self-reported outcomes (De Vita et al., 2016). In this study, we selected multiple large publicly available datasets composed of 100 LPS tumor samples and 21 adipose tissues. We have discovered four genes of prognostic value (CENPF, FOXM1, MCM4, and TOP2A), and further prioritized MCM4 as a key biomarker of LPS associated with tumor invasiveness (tumor stage, grade, and Ki67 labeling index) and prognostication. These findings were validated by the data registries from Oncomine as well as the clinicopathological data from our institution. However, the underlying mechanism of MCM4 related to a worse prognosis remains unclear. Previous literature has suggested the MCM gene as a direct index of tumor (Choy et al., 2016) and replicative stress responder of genome instability (Ibarra et al., 2008). MCMs have also been implicated in the epithelial–mesenchymal transition (Zhang et al., 2019) and other well-known cancer cell signaling pathways, such WNT, CDK, MYCN, etc. (Shohet et al., 2002; Yu et al., 2020). More interestingly, we found that such prognostic significance of MCM4 could be further expanded to the pan-sarcoma population at a broader scale, and the association of genomic instability and HR deficiency (BRCAness) with MCM4 expression might be a common genomic and transcriptomic portrait shared among different sarcoma histologies. To our knowledge, there are no studies reporting such prognostic significance of MCM4 and its associated genomic/transcriptomic signature in LPS as well as in STS.

Genome instability and HR deficiency have been recently postulated as key molecular characteristics of dysregulated DNA repair pathways in STS with potential therapeutic implications. In addition to the traditional knowledge of BRCAness as the Achilles’ heel of cancer cells to PARPi and platinum-based chemotherapy, it is suggested that DNA-damaging agents could aggravate the copy number aberration in the chromosomal unstable tumor, surpassing the tolerance limit of the genome and leading to tumor cell death (Andor et al., 2017). Besides, targeting the HR and non-homologous end-joining (NHEJ) mechanism of cancer cell might further enhance such therapeutic sensitivity of the cancer cells with high levels of CNVs (Gregg et al., 1993; Zhang et al., 2014). However, previous clinical trials of PARPi and DNA-damaging agents mostly failed to confirm such vulnerability in unselected sarcoma population (Kalofonos et al., 2004; Choy et al., 2014). Interestingly, our study demonstrated that genomic instability and BRCAness phenotype could vary tremendously, both inter- and intra- in STS subtypes, which were correlative with tumoral MCM4 expression. On the basis of such observations, we speculated that DNA repair defect-targeted therapies might be implicated for MCM4-high subset, rather than the total population, of STS. Surprisingly, in accordance with our hypothesis, the therapeutic exploitation assay of PTCCs in our study clearly showed that the MCM4-high subset of STS owns a remarkably higher sensitivity to cisplatin treatment than MCM-low tumors. These findings warrant further elucidation of MCM4 as a biomarker for patient-tailored management of STS using DNA-damaging chemotherapy. The activity of PARPi and PARPi/chemotherapy combination therapy in MCM4-high STS is an even more attractive potential, although requiring more translational as well as mechanistic investigations in the future.

## Data Availability Statement

The datasets presented in this study can be found in online repositories. The names of the repository/repositories and accession number(s) can be found in the article/Supplementary Material.

## Ethics Statement

The studies involving human participants were reviewed and approved by Ruijin Hospital Ethics Committee. The patients/participants provided their written informed consent to participate in this study.

## Author Contributions

QL, QB, and YX conducted experiments and analyzed the data and wrote the manuscript. YF, ZJ, and JW helped with the experiments. WZ and YS were co-corresponding authors. All authors contributed to the article and approved the submitted version.

## Conflict of Interest

The authors declare that the research was conducted in the absence of any commercial or financial relationships that could be construed as a potential conflict of interest.
