# Supplementary material for: MCM4 Is a Novel Biomarker Associated With Genomic Instability, BRCAness Phenotype, and Therapeutic Potentials in Soft-Tissue Sarcoma
Source: Front Cell Dev Biol. 2021 Jun 10;9:666376. doi: 10.3389/fcell.2021.666376 (PMC8222794; doi:10.3389/fcell.2021.666376)
Supplement: Supplementary file 1 [file Data_Sheet_1.doc]

Supplementary Material

**Table S1.** Basic information of database used in our study.

| **Database** | **Web link** | **Citation** |
| --- | --- | --- |
| GEO | <https://www.ncbi.nlm.nih.gov/gds/> | (Clough and Barrett, 2016) |
| STRING | <https://string-db.org/> | (Szklarczyk et al., 2015) |
| TCGA | <https://portal.gdc.cancer.gov/> | (Cancer Genome Atlas Research Network. Electronic address and Cancer Genome Atlas Research, 2017) |
| Oncomine | <https://www.oncomine.org/> | (Rhodes et al., 2007) |
| UCSC Xena | <https://xenabrowser.net/datapages/> | (Goldman et al., 2020) |
| cBioPortal | <http://www.cbioportal.org/> | (Cerami et al., 2012) |
| GTEx | <https://commonfund.nih.gov/GTex> | (Consortium, 2020) |

**Abbreviations:** GEO, Gene Expression Omnibus; STRING, search tool for recurring instances of neighbouring genes; TCGA, The Cancer Genome Atlas; GTEx, Genotype-Tissue Expression.

**Table S2. The clinicopathological data of 8 STS patients.**

| **Patient** | a | b | c | d | e | f | g | h |
| --- | --- | --- | --- | --- | --- | --- | --- | --- |
| **Sex** | Female | Male | Female | Male | Female | Male | Female | Female |
| **Age** | 54 | 37 | 52 | 76 | 35 | 53 | 85 | 62 |
| **Type** | UPS | LPS | LMS | LPS | ES | LPS | MFS | UPS |
| **Location** | Thigh | Thigh | Armpit | Thigh | Thigh | Thigh | Thigh | Calf |
| **MCM4** | Low | Low | Low | Low | High | High | High | High |
| **Ki-67** | 5% | 2% | 50% | 20% | 40% | 30% | 90% | 90% |
| **MDM-2** | ND | (+) | ND | (+) | ND | (+) | (+) | ND |
| **CDK-4** | (-) | (+) | ND | (+) | ND | (-) | (+) | ND |
| **S-100** | (-) | (+) | (-) | (-) | (-) | (+) | (-) | (-) |
| **CD34** | (-) | (-) | (-) | (-) | (-) | (+) | (+) | (+) |

**Abbreviations:** ND, not determined; LPS, Liposarcoma; LMS, Leiomyosarcoma; MFS, Myxofibrosarcoma; UPS, Undifferentiated pleomorphic sarcoma; ES, Epithelioid sarcoma; MCM4, mini-chromosome maintenance protein 4; MDM-2, murine double minute 2; CDK-4, cyclin dependent kinase 4.

**Table S3. The primary and secondary antibodies for Western blotting.**

| **Antibody** | **Brand (cat. number)** | **Dilution** |
| --- | --- | --- |
| Rabbit polyclonal antibody to Phospho-Akt | CST (cat. 4060) | 1: 2000 |
| Rabbit polyclonal antibody to Akt | CST (cat. 4685) | 1: 1000 |
| Rabbit polyclonal antibody to Phospho-S6 | CST (cat. 2211) | 1: 1000 |
| Rabbit polyclonal antibody to S6 | CST (cat. 2217) | 1: 1000 |
| Rabbit polyclonal antibody to MCM4 | CST (cat. 12973) | 1: 1000 |
| Rabbit polyclonal antibody to β-Actin | CST (cat. 4970) | 1: 1000 |
| Goat anti-rabbit IgG secondary antibody | CST (cat. 7074) | 1: 1000 |


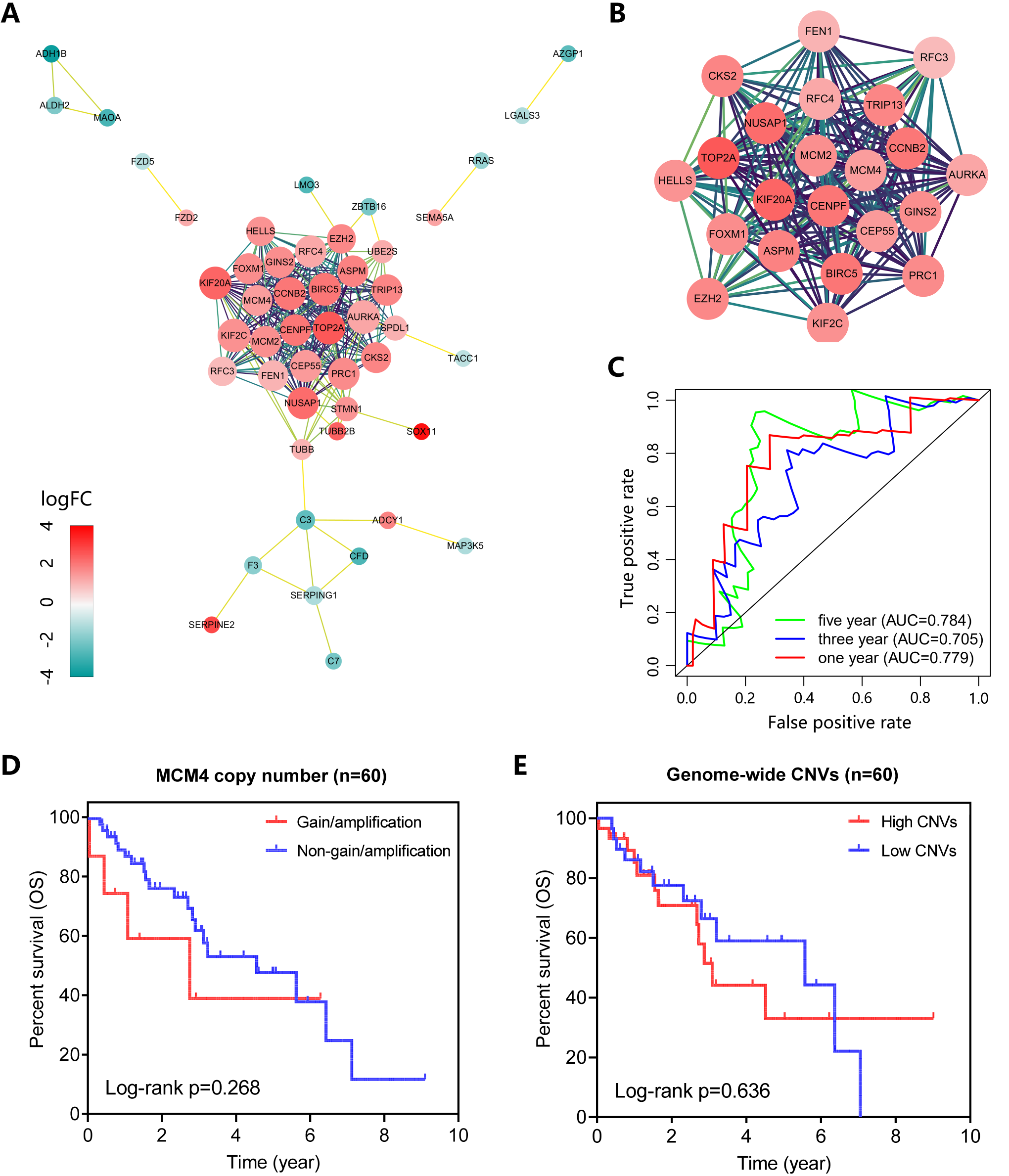


**Figure S1.** **MCM4 as a robust predictor of the survival in LPS patients.** (A) Protein-protein interaction network of common DEGs. The color of the nodes and edges indicated gene expression levels and their co-expression scores, respectively. The size of each node was proportional to its degree (the number of connections). (B) Prime module from the PPI network. Module analysis identified 22 central nodes as the hub genes. (C) Predictive value of the survival outcome by the MCM4 expression demonstrated by the ROC curves. (D-E) Kaplan-Meier curves of the patients in relation to the MCM4 copy numbers and genome-wide CNVs of the samples (Log-rank test p>0.05).


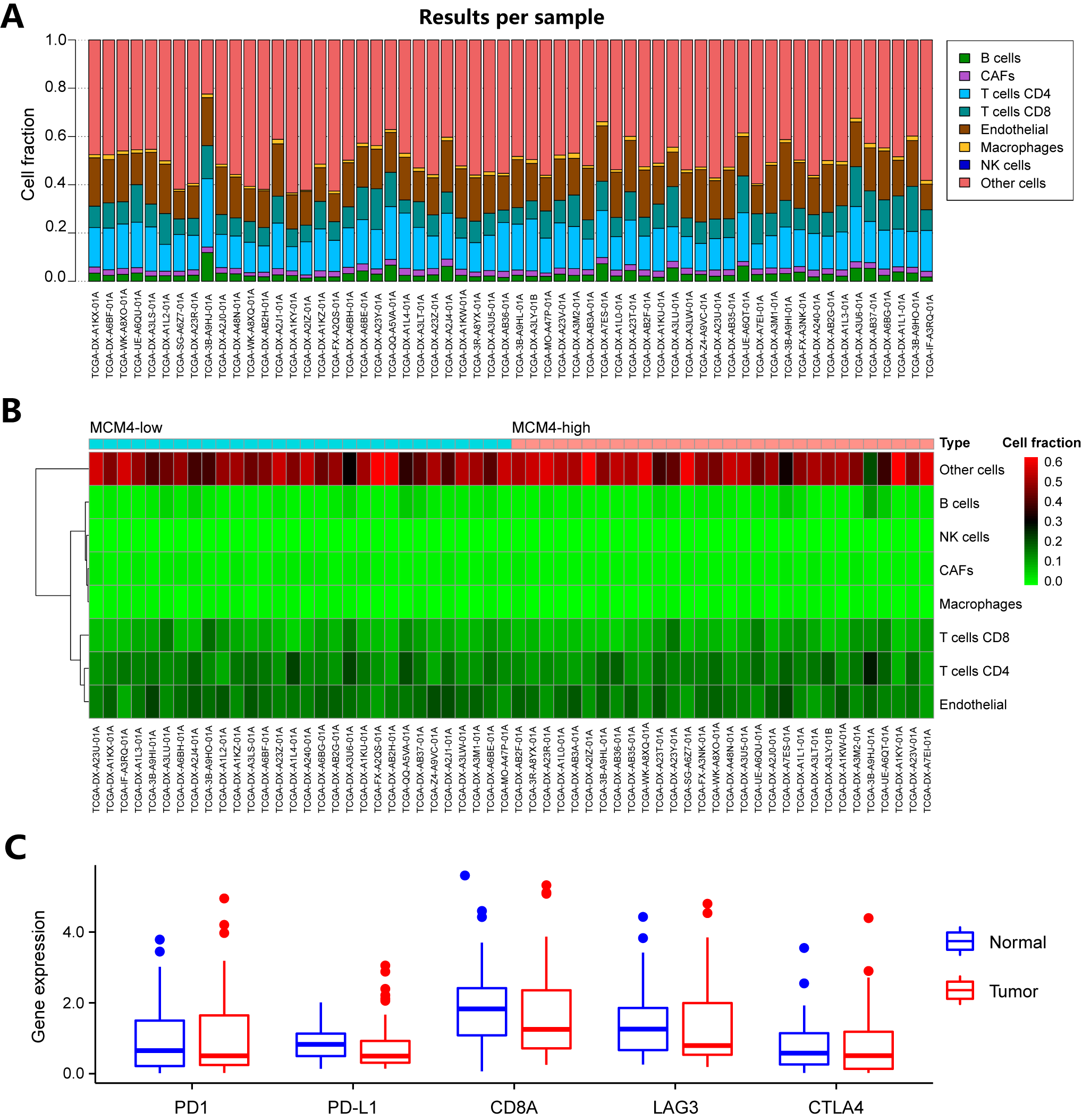


**Figure S2. The immunological differences between MCM4-high and -low LPS.** (**A**) Immune cells proportion calculated for the 60 LPS samples by EPIC software. (**B**) Correlation of MCM4 expression with the percentage of immune cells. (**C**) Comparison of immune checkpoint molecule expression (PD-1, PD-L1, CD8A, LAG3, and CTLA4) between MCM4-high and -low subsets (Wilcoxon test, p>0.05, n=60).


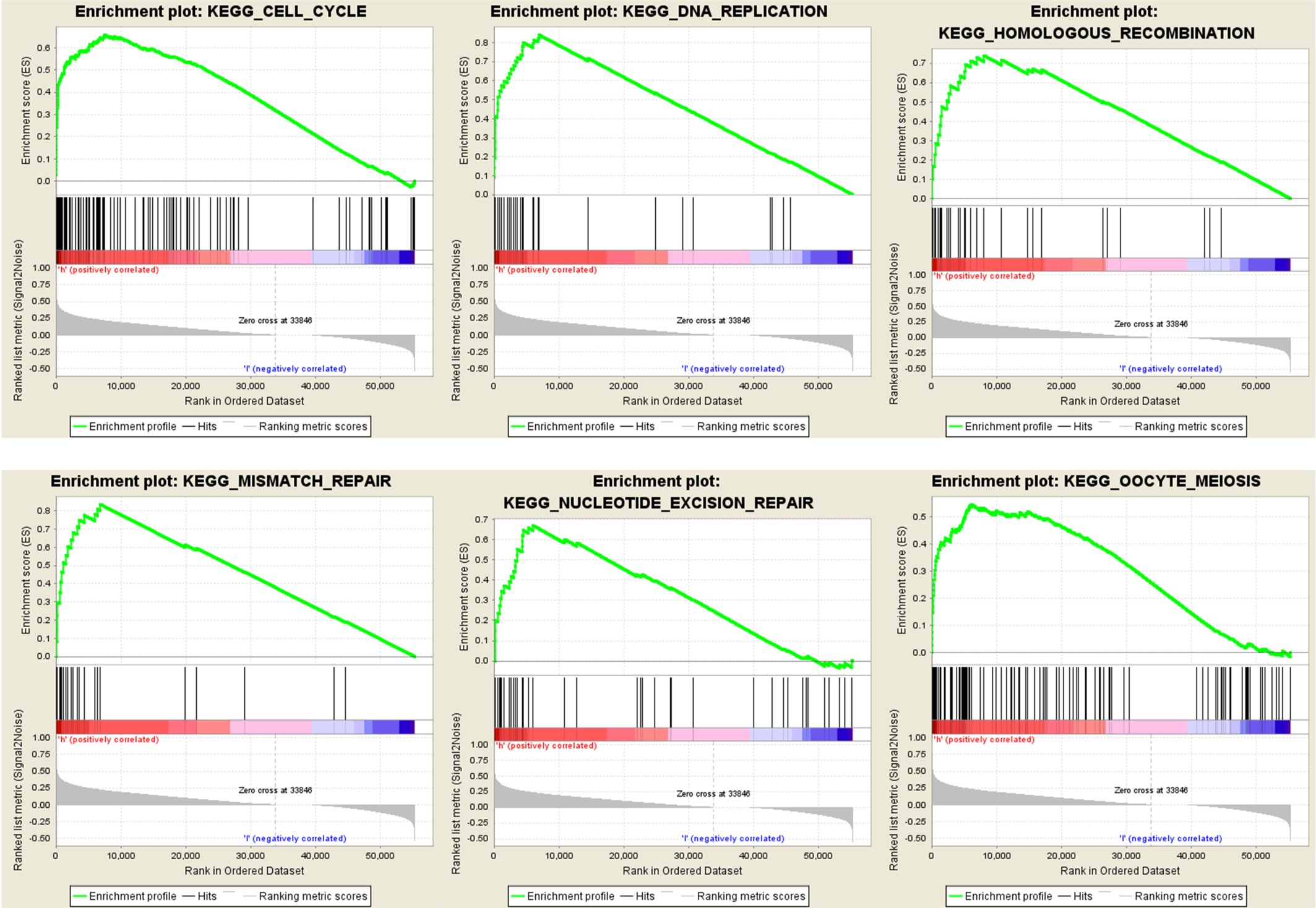


**Figure S3**. The KEGG pathways that were significantly enriched in MCM4-high versus the MCM4-low subset of LPS, presented as the raw enrichment plot corresponding to Figure 2E.


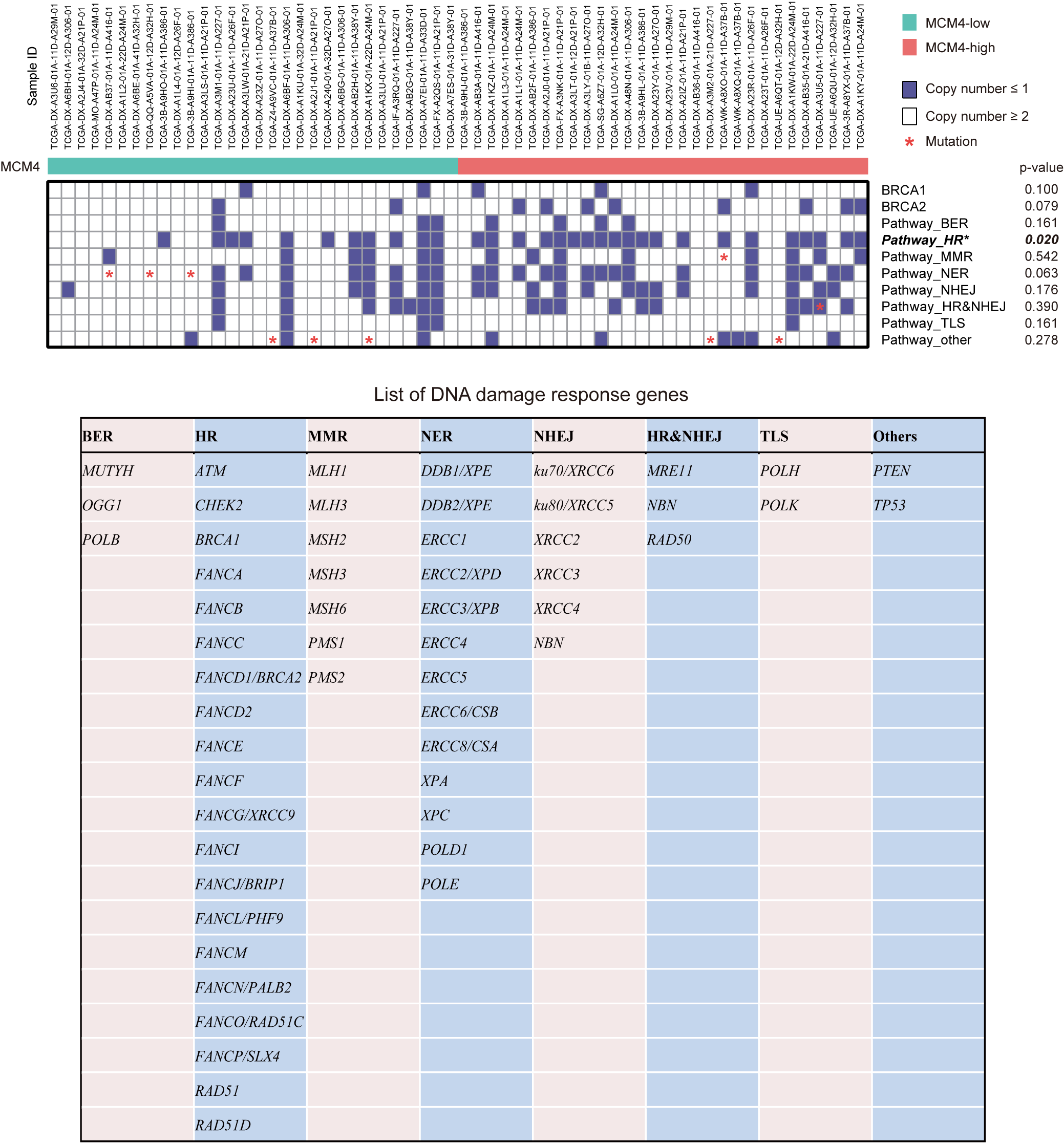


**Figure S4.** MCM4-high LPS more frequently harbored copy number loss in genes of HR pathway (permutation test, p=0.020, n=60).


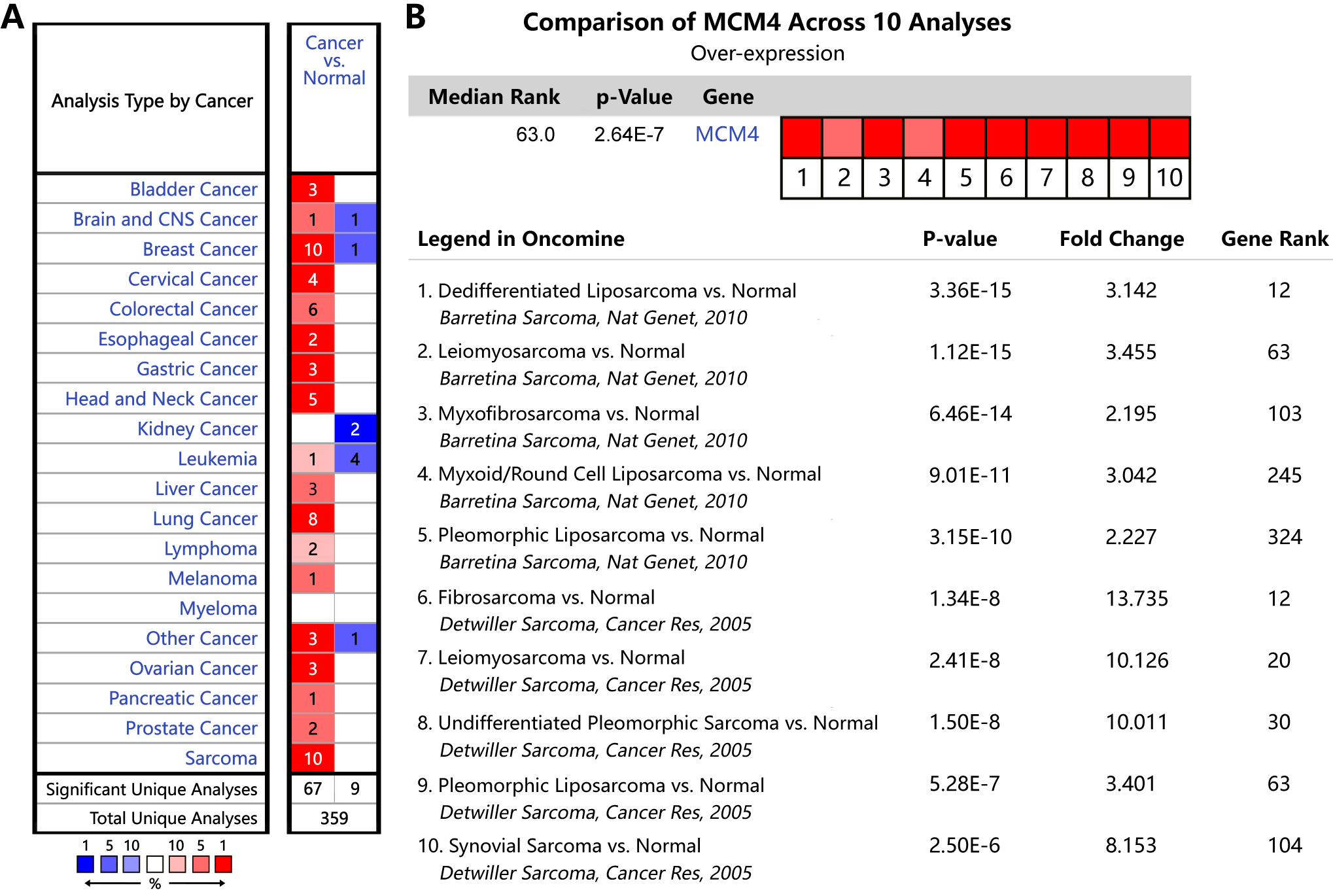


**Figure S5**. **Validation of MCM4 as a biomarker of STS by Oncomine database.** (A) MCM4 expression status in all cancer studies. The best gene rank percentile determines cell color, where red represents overexpression, and blue represents decreased expression. The numbers in the cells represent the number of studies. As shown, a total of 10 STS registries supporting the high tumoral expression of MCM4 versus normal. (B) MCM4 was found to be consistently overexpressed in LPS, leiomyosarcoma, fibrosarcoma, undifferentiated pleomorphic sarcoma, and synovial sarcoma.


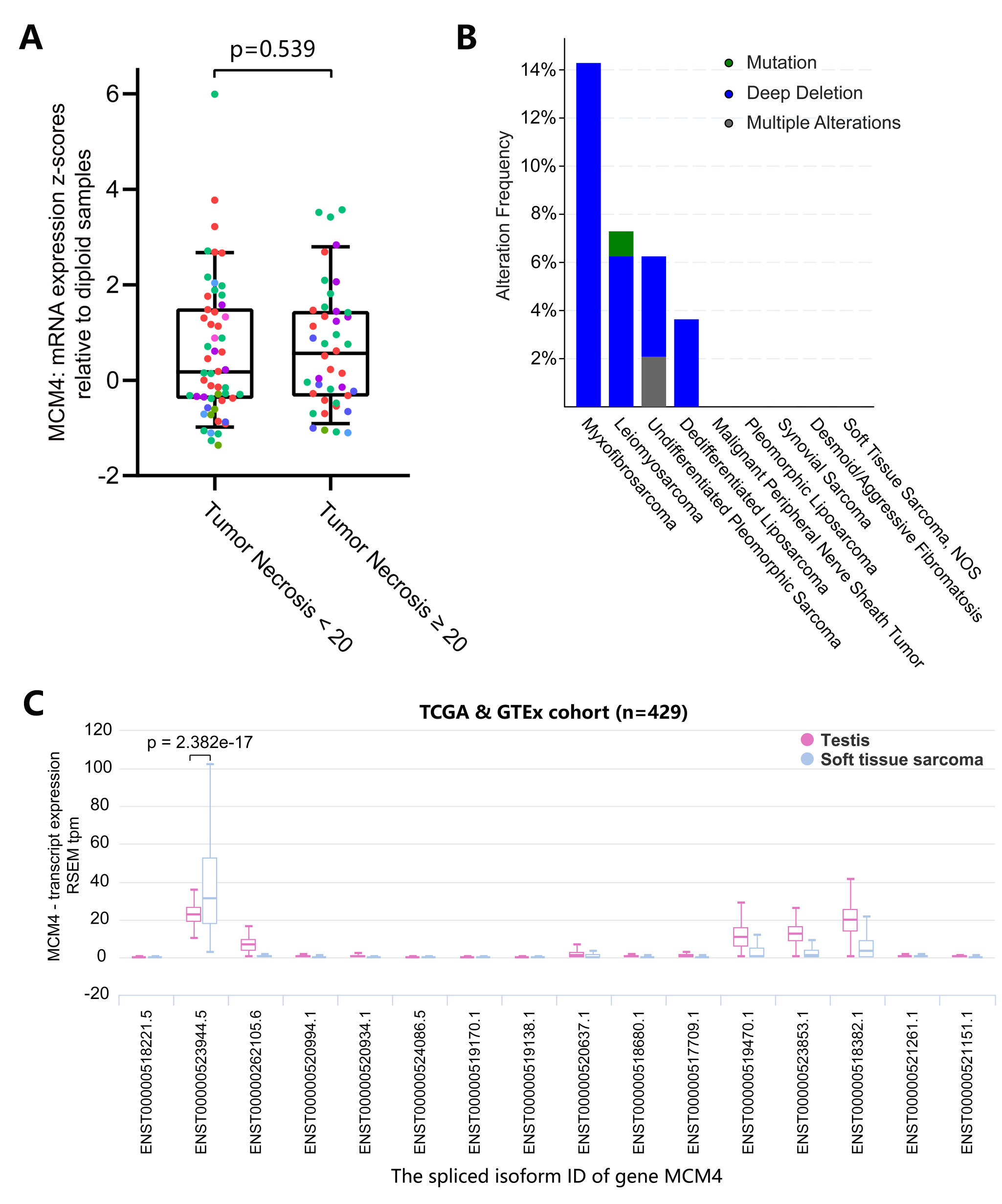


**Figure S6. Correlation between MCM4 expression and other parameters.** (A) MCM4 showed no relevance with percent necrosis (Wilcoxon test, p=0.539, n=54). (B) HR mutation versus histology subtype in TCGA database. (C) Expression of the spliced isoform of MCM4 in soft-tissue sarcoma versus testis.

**Reference**

Cancer Genome Atlas Research Network. Electronic address, e.d.s.c., and Cancer Genome Atlas Research, N. (2017). Comprehensive and Integrated Genomic Characterization of Adult Soft Tissue Sarcomas. Cell *171*, 950-965 e928.

Cerami, E., Gao, J., Dogrusoz, U., Gross, B.E., Sumer, S.O., Aksoy, B.A., Jacobsen, A., Byrne, C.J., Heuer, M.L., Larsson, E.*, et al.* (2012). The cBio cancer genomics portal: an open platform for exploring multidimensional cancer genomics data. Cancer Discov *2*, 401-404.

Clough, E., and Barrett, T. (2016). The Gene Expression Omnibus Database. Methods Mol Biol *1418*, 93-110.

Consortium, G.T. (2020). The GTEx Consortium atlas of genetic regulatory effects across human tissues. Science *369*, 1318-1330.

Goldman, M.J., Craft, B., Hastie, M., Repecka, K., McDade, F., Kamath, A., Banerjee, A., Luo, Y., Rogers, D., Brooks, A.N.*, et al.* (2020). Visualizing and interpreting cancer genomics data via the Xena platform. Nat Biotechnol *38*, 675-678.

Rhodes, D.R., Kalyana-Sundaram, S., Mahavisno, V., Varambally, R., Yu, J., Briggs, B.B., Barrette, T.R., Anstet, M.J., Kincead-Beal, C., Kulkarni, P.*, et al.* (2007). Oncomine 3.0: genes, pathways, and networks in a collection of 18,000 cancer gene expression profiles. Neoplasia *9*, 166-180.

Szklarczyk, D., Franceschini, A., Wyder, S., Forslund, K., Heller, D., Huerta-Cepas, J., Simonovic, M., Roth, A., Santos, A., Tsafou, K.P.*, et al.* (2015). STRING v10: protein-protein interaction networks, integrated over the tree of life. Nucleic Acids Res *43*, D447-452.
